# Supplementary figures and images for: Adverse Effects of Intravesical OnabotulinumtoxinA Injection in Patients with Idiopathic Overactive Bladder or Neurogenic Detrusor Overactivity: A Systematic Review and Meta-Analysis of Randomized Controlled Studies
Source: Toxins (Basel). 2024 Aug 5;16(8):343. doi: 10.3390/toxins16080343 (PMC11359369; doi:10.3390/toxins16080343)

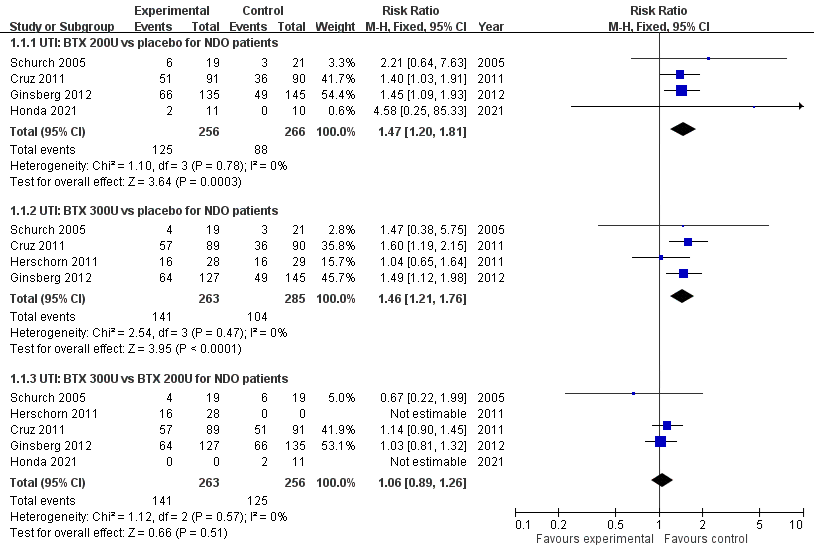

Supplement: Supplementary file 1 [file toxins-16-00343-s001.zip › Supplement 1a.png]

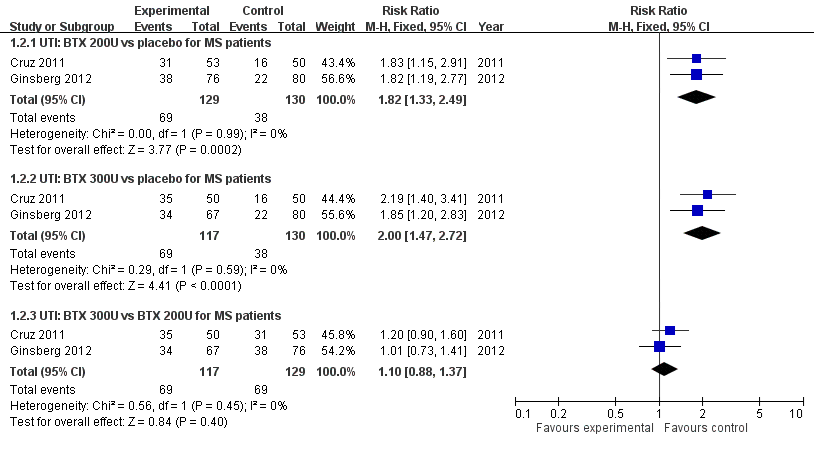

Supplement: Supplementary file 1 [file toxins-16-00343-s001.zip › Supplement 1b.png]

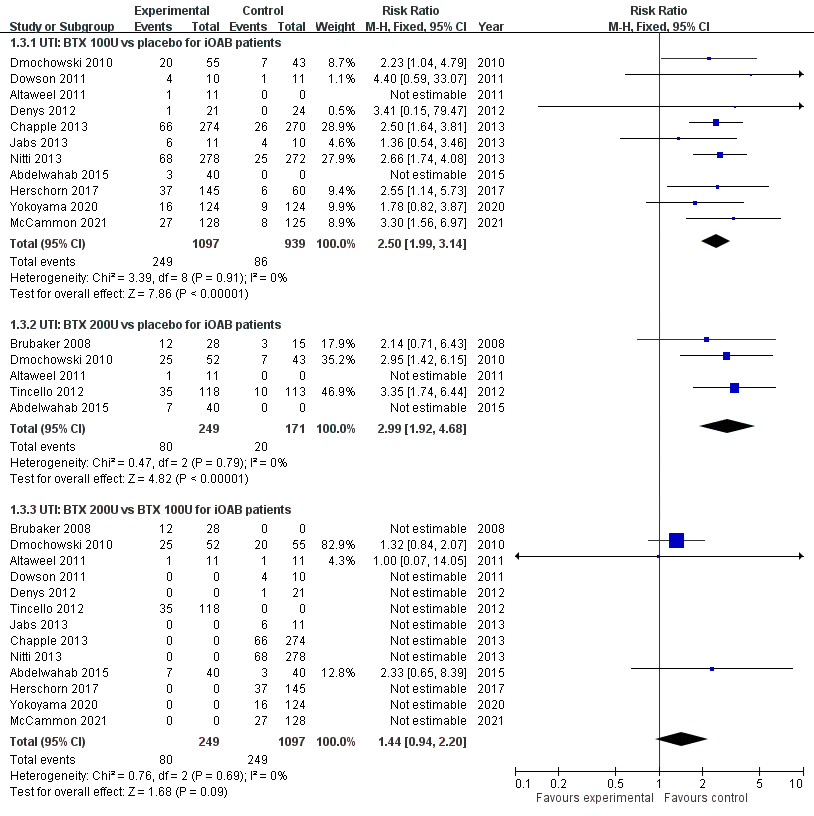

Supplement: Supplementary file 1 [file toxins-16-00343-s001.zip › Supplement 1c.png]

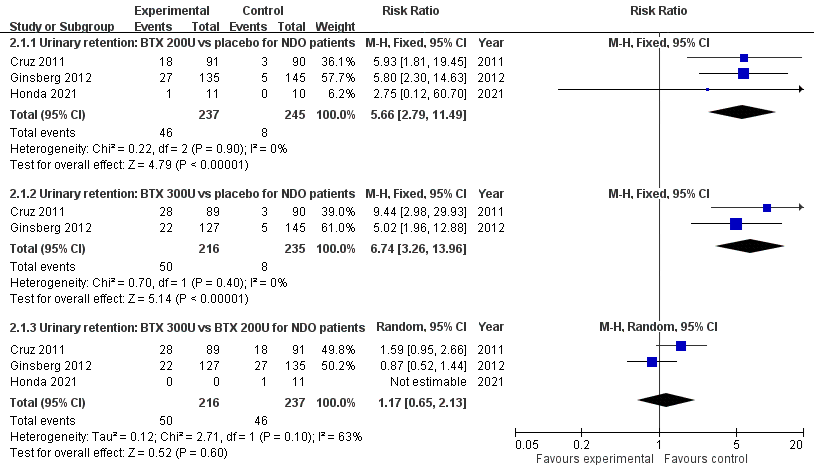

Supplement: Supplementary file 1 [file toxins-16-00343-s001.zip › Supplement 2a.png]

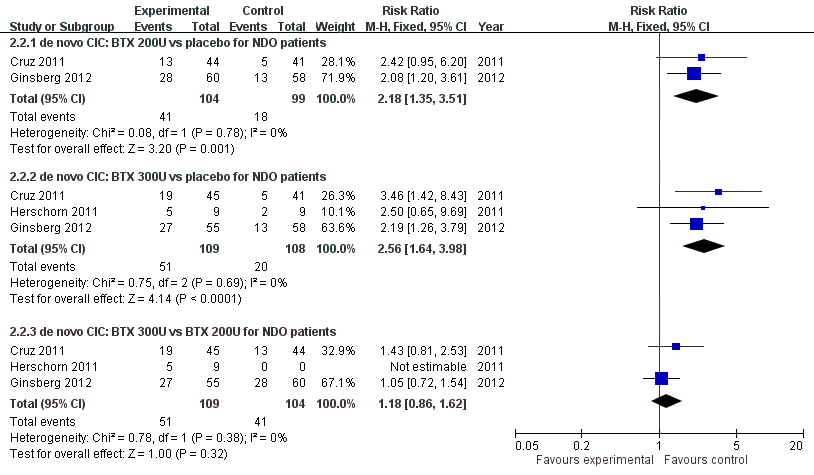

Supplement: Supplementary file 1 [file toxins-16-00343-s001.zip › Supplement 2b.png]

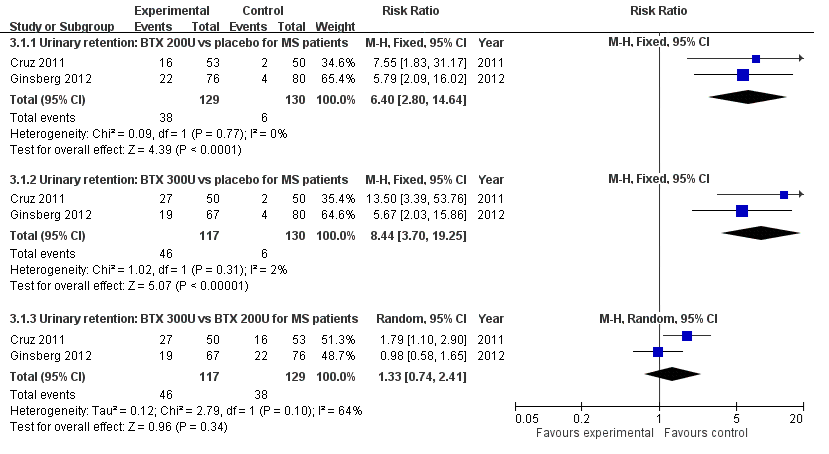

Supplement: Supplementary file 1 [file toxins-16-00343-s001.zip › Supplement 3a.png]

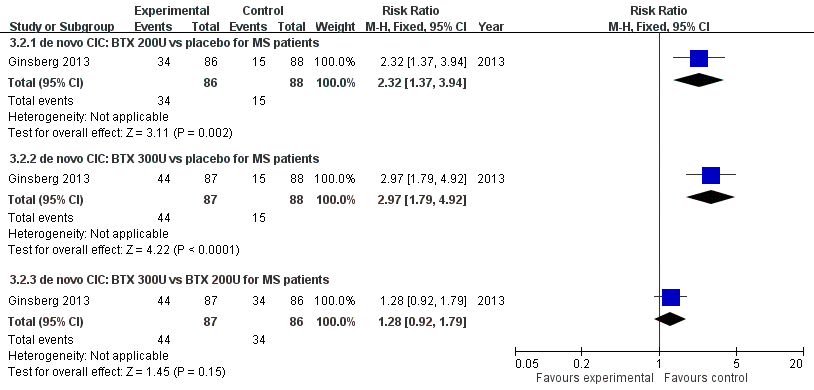

Supplement: Supplementary file 1 [file toxins-16-00343-s001.zip › Supplement 3b.png]

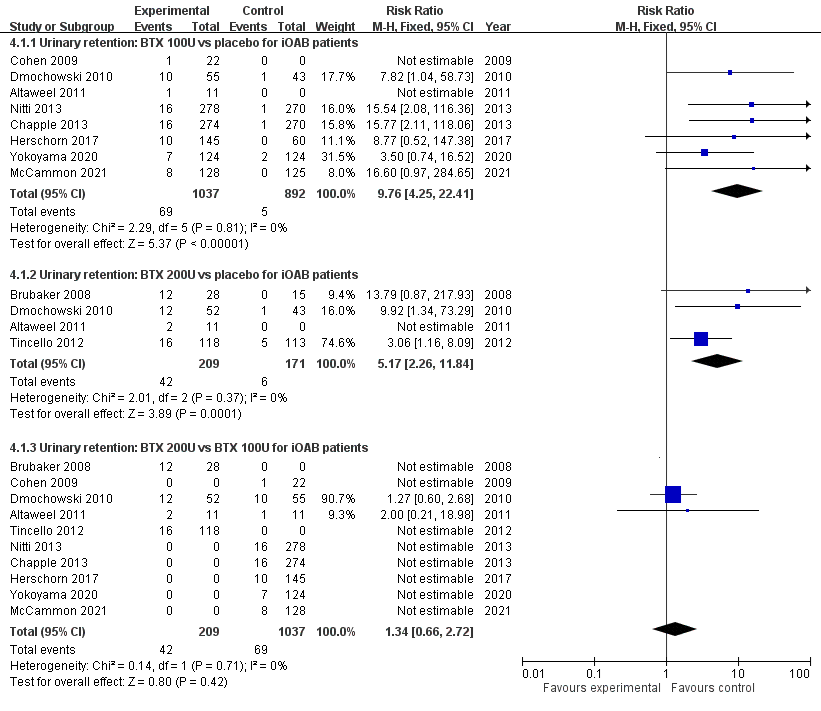

Supplement: Supplementary file 1 [file toxins-16-00343-s001.zip › Supplement 4a.png]

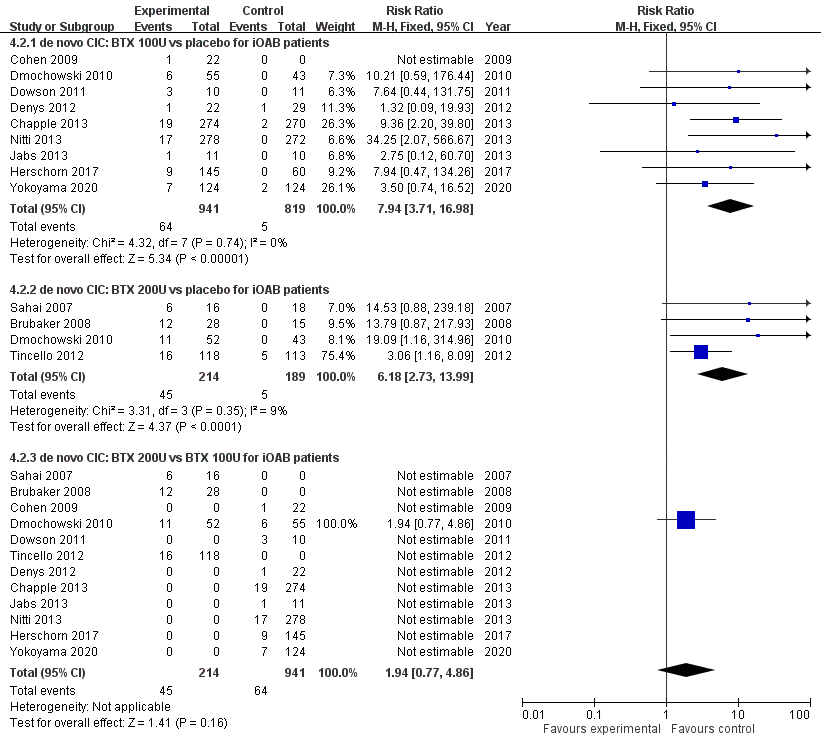

Supplement: Supplementary file 1 [file toxins-16-00343-s001.zip › Supplement 4b.png]

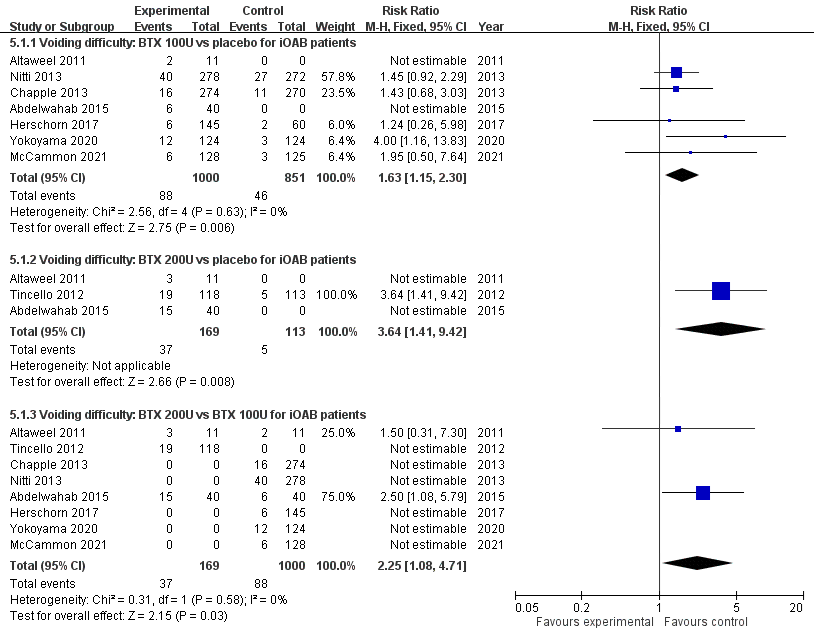

Supplement: Supplementary file 1 [file toxins-16-00343-s001.zip › Supplement 5.png]

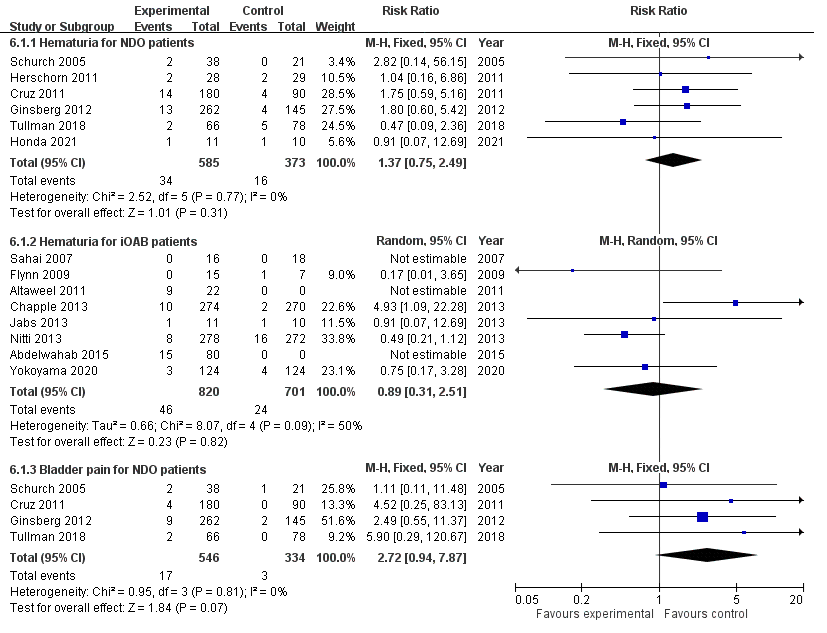

Supplement: Supplementary file 1 [file toxins-16-00343-s001.zip › Supplement 6.png]

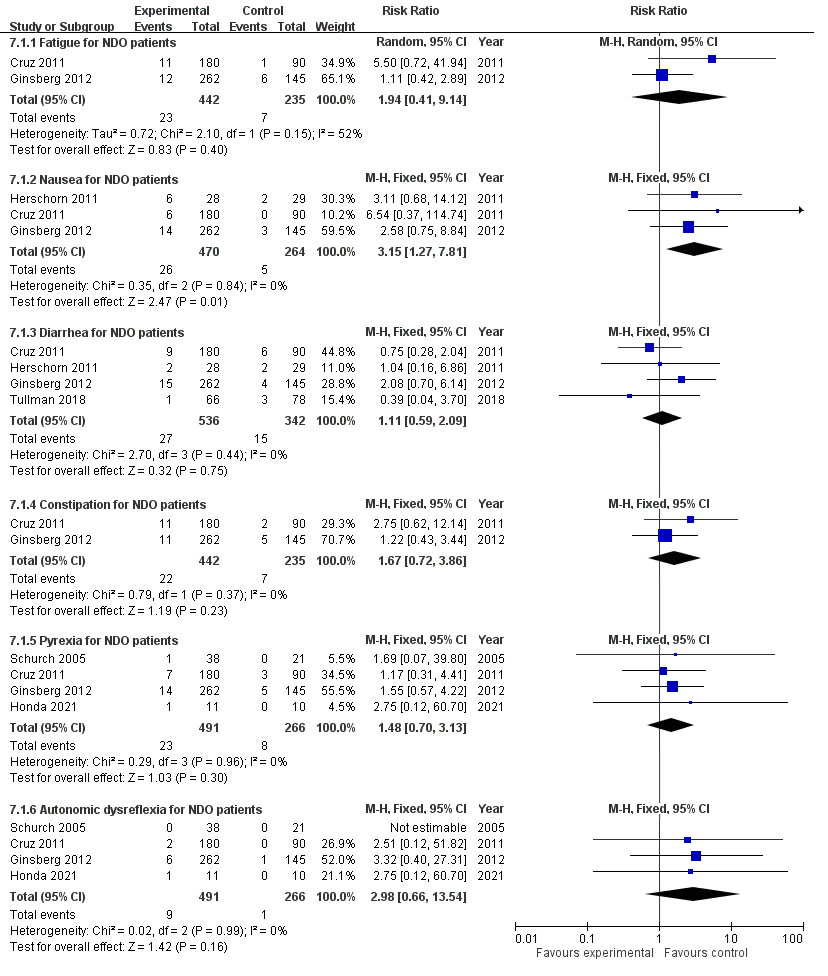

Supplement: Supplementary file 1 [file toxins-16-00343-s001.zip › Supplement 7.png]
